# A Facial Recognition Mobile App for Patient Safety and Biometric Identification: Design, Development, and Validation

**DOI:** 10.2196/11472

**Published:** 2019-04-08

**Authors:** Byoungjun Jeon, Boseong Jeong, Seunghoon Jee, Yan Huang, Youngmin Kim, Gee Ho Park, Jungah Kim, Maierdanjiang Wufuer, Xian Jin, Sang Wha Kim, Tae Hyun Choi

**Affiliations:** 1 Interdisciplinary Program for Bioengineering Graduate School Seoul National University Seoul Republic of Korea; 2 Oezsoft Inc Seoul Republic of Korea; 3 Interdisciplinary Program in Stem Cell Biology College of Medicine Seoul National University Seoul Republic of Korea; 4 Department of Chemistry Seoul National University College of Natural Sciences Seoul Republic of Korea; 5 Department of Plastic and Reconstructive Surgery Seoul National University College of Medicine Seoul Republic of Korea; 6 Institute of Human Environment Interface Biology Department of Plastic and Reconstructive Surgery Seoul National University College of Medicine Seoul Republic of Korea

**Keywords:** facial recognition, patient identification systems, biometric identification, patient safety, smartphone, mobile applications

## Abstract

**Background:**

Patient verification by unique identification is an important procedure in health care settings. Risks to patient safety occur throughout health care settings by failure to correctly identify patients, resulting in the incorrect patient, incorrect site procedure, incorrect medication, and other errors. To avoid medical malpractice, radio-frequency identification (RFID), fingerprint scanners, iris scanners, and other technologies have been implemented in care settings. The drawbacks of these technologies include the possibility to lose the RFID bracelet, infection transmission, and impracticality when the patient is unconscious.

**Objective:**

The purpose of this study was to develop a mobile health app for patient identification to overcome the limitations of current patient identification alternatives. The development of this app is expected to provide an easy-to-use alternative method for patient identification.

**Methods:**

We have developed a facial recognition mobile app for improved patient verification. As an evaluation purpose, a total of 62 pediatric patients, including both outpatient and inpatient, were registered for the facial recognition test and tracked throughout the facilities for patient verification purpose.

**Results:**

The app was developed to contain 5 main parts: registration, medical records, examinations, prescriptions, and appointments. Among 62 patients, 30 were outpatients visiting plastic surgery department and 32 were inpatients reserved for surgery. Whether patients were under anesthesia or unconscious, facial recognition verified all patients with 99% accuracy even after a surgery.

**Conclusions:**

It is possible to correctly identify both outpatients and inpatients and also reduce the unnecessary cost of patient verification by using the mobile facial recognition app with great accuracy. Our mobile app can provide valuable aid to patient verification, including when the patient is unconscious, as an alternative identification method.

## Introduction

### Background

For patient safety, the minimum requirements for patient identification in a hospital setting include 2 different patient-provided verifications that usually include patient name, the registered patient number, or date of birth [1-3]. Every visit to any hospital location for examination, treatment, or other health care services requires patient identification.

The purpose of patient verification is to minimize the potential for medical malpractice and other risks to patient safety. Several incidents because of lack of patient identification during treatment have been reported. In 2014, a physician got confused with 2 patients’ surnames and gave the wrong patient’s name to the surgeon over the phone, which resulted in heart surgery on the wrong patient [4]. In 2000, the New York State Health Department cited Staten Island University Hospital for failing to monitor and discipline its chief of neurosurgery, who, according to the state, had operated on the wrong side of a patient’s brain for the second time in 5 years [5]. It was because of lack of confirmation on all the necessary information, in this case, computed tomography (CT) scans, before making an incision on the left cerebellum. The case could be prevented if all the information of the patient needed before surgery was confirmed.

Furthermore, in 1994, a similar error occurred in a lung cancer patient in Texas [6]. The surgeon admitted that he negligently removed the wrong lung, and it was discovered 1 week after the surgery when the patient himself found this after reviewing his medical records. These cases could be easily prevented by surgeons or other faculties by identifying the patient and checking all the necessary information before the operation. According to the Joint Commission, the number of reported sentinel events in 2017 was 400, which is only a small fraction of actual events [7].

To resolve the current issues with patient verification, some hospitals have adapted newer technology, such as disposable, scannable radio-frequency identification (RFID) bracelets, for more precise and easier patient verification [8-11]. As the typical identification method is to ask patients for their name; patient number, which is a complicated series of numbers; or other appropriate information, it is impossible to verify a patient when they are unconscious or when they are not able to remember them. The RFID bracelet, which can simply be scanned, facilitates an improved verification process. However, the limitation of employing physical objects such as RFID bracelets, cards, tokens, or keys is that the object must always be presented and kept secure [10,12,13]. In other words, if the physical objects such as RFID bracelets are lost or unable to scan, patient identification is not possible with the objects. Therefore, biometric measure, which represents an alternative method for patient verification without the necessity for physical objects, has emerged.

Biometrics refers to the recognition of individuals based on their anatomical, physiological, and/or behavioral characteristics, which permits identification without physical objects. Biometric options are not limited to fingerprint scanners but also include palm scanners, iris scanners, etc for patient verification [14,15]. However, there are limitations and disadvantages associated with these measures. Fingerprint collection requires patients to physically place their finger onto the scanner every time, which may facilitate disease transfer through physical contact with the scanner. Disease transfer through fingerprint includes a risk of transfer of infectious microorganisms that are enteric and respiratory pathogens [16]. Furthermore, unconscious patients are not able to place their finger onto the scanner by themselves. Although iris scans seem to be the ideal option for patient verification compared with other various verification methods, the main drawback is that it is difficult to scan the iris of an unconscious patient, and therefore, it is virtually impossible to verify unconscious patients with an iris scan [15,17]. Furthermore, when a surgical operation is required, patients must be put under anesthesia, negating the iris scanner as a valid option.

One interesting biometric is facial recognition, which does not have the disadvantages of the other biometrics options described above. Facial recognition does not require any physical contact with a device for recognition, and patients can be recognized even when they are unconscious. The comparison of facial characteristics obtained from a patient with stored or preregistered facial records in a database allows the recognition process to verify the patient. Previous research using Microsoft Kinect v2 sensor for patient verification demonstrated that facial features could be implemented for patient verification [18]. Furthermore, other prior work revealed that the CT scans can be used as facial recognition with moderately high accuracy [19]. However, certain sensors and specific actions were required to acquire and process facial information in prior studies. In addition, facial recognition systems require simple and direct processes to be useful in identifying patients in various conditions and hospital settings.

### Objectives

Here, we have applied a facial recognition system to develop a hospital-friendly mobile app for patient verification. We evaluated the performance of the mobile app on a total of 62 hospital inpatients and outpatients. The aim of this study was to see if the performance of the mobile app is suitable in a hospital setting as an alternative method for patient verification.

## Methods

### The Facial Recognition System–Embedded Mobile App

We developed an Android-based app in the Eclipse environment using Java (Oracle Corporation). A facial recognition engine powered by Oezsoft Inc was modified to leverage the Native Development Kit (NDK) library and was used in Java through the Java Native Interface. The NDK library was developed in Linux using a GNU Compiler Collection compiler. A mobile camera captures the characteristics of individuals’ facial features as if a mobile phone was used to photograph the individual’s face. The facial recognition engine extracts 27 major landmarks from the 2-dimensional facial image captured by the mobile camera. Then, the 3-dimensional facial learning data are used to compile the 3-dimensional portrait by extracting the major landmarks to create a comparable facial template, which is then registered in the mobile phone.

### Mobile App Interface Design

The interface is designed to be easy to use and familiar to medical staff because the facial data collection time is short, and the patient would want to move quickly. The interface of the app contains 5 main recording menus: the registration, medical records, examinations, prescriptions, and appointments.

#### Registration

The registration part contains facial information acquiring step and basic personal information recording step to register a patient on the app environment. With a mobile camera in operation, the facial data are extracted from the image, which is stored in a 3-dimensional vector form and is used as a facial template. The basic personal information such as the name, date of birth, sex, and phone number was recorded in the app.

#### Medical Records

The medical record part contains selecting a department, outpatient, inpatient, and surgery. As each option was selected, the medical recording part is ready to be entered. Examination and prescription options were also selected for later review by other medical staff. All the records were uploaded to the app system and the hospital’s electronic medical record (EMR) separately because the systems are not interworking yet. The records in the app were compared against the information recorded in the EMR to see if the information in the app is preserved throughout the facilities and matches with the EMR. After the facial recognition test of each patient in each facility, the recorded information in the app was compared with the EMR as a preliminary test for future application and EMR interlocking system.

#### Examinations and Prescriptions

The examination and prescription parts show whether the patient has been to an ordered facility for examination or prescription. A medical staff at the facility can check whether the examination or prescription has been done. For instance, the examination list shows a red mark if the ordered examination or prescription has not been done and green mark if the ordered examination or prescription has been done.

#### Appointments

The appointments let the medical staff arrange the next schedule on the app so that the different medical staff can see the record when the patient arrives for the next visit. The listed options for the appointment are outpatient, inpatient, surgery, and the list of departments the patient would visit.

### Ethics Statement

This project was approved by the Institutional Review Board (IRB) of Seoul National University Hospital (IRB approval number: 1701-027-821). All experiments dealing with human or human products were conducted with informed consent and carried out in accordance with the relevant guidelines and regulations. Every patient and their parents were told about the facial recognition process of how the verification using a mobile facial app would be used for their identification. After giving them detailed information about identification, permission from both patients and their parents were obtained by signing the informed consent form.

### Outpatient Studies

Medical staff from the plastic surgery department of Seoul National University Children’s Hospital were instructed to generate facial profiles of registered outpatient plastic surgery patients by taking pictures with the provided mobile device. All patients were first verified using routine verification methods such as matching patient number or name. Then, a medical staff registered patients for facial recognition upon admission. When a patient entered the examination room of the outpatient clinic, the patient was then verified using the facial recognition mobile app. The mobile app was used as a secondary method to verify the patient and to record patient information, necessary examinations, prescription information, and to make a reservation for the patient’s next visit. When the patient moved through the facility to additional examination or treatment rooms, medical staff used the facial recognition mobile app to verify the patient and to determine the required examinations and/or treatments. Facility visits were recorded, which indicates the number of different facilities where each patient was verified by both traditional identification and the facial recognition mobile app. Each time the facial recognition mobile app was operated to verify a patient, it was recorded as pass verification when the mobile app identified the patient correctly, otherwise it was recorded as fail verification. Male and female verification numbers were counted, which indicates the sum of facility visits by each patient for whom facial verification was performed. A total of 30 outpatients were all pediatric patients, and the patients were aged between 1 and 13 years. All patients were conscious because they only visited the hospital for diagnostic or preoperative examination purposes.

### Inpatient Studies

Inpatients scheduled for surgical operation in the plastic surgery department as well as intensive care unit patients were also selected for facial verification. Similar to outpatients described above, hospital faculty verified inpatients first with the routinely used patient verification method and then with facial recognition mobile app at the entrance of the operating room, in the operating room, and in the recovery room. After each facial recognition verification, patients were treated, examined, or prescribed medication. As stated in the outpatient section above, facility visits and pass or fail facial verification numbers were recorded separately. Facility visits indicate the number of different facilities visited by each patient. If the patient was correctly identified at the facility, it was recorded as a pass verification, and if the patient was identified incorrectly, it was recorded as a fail verification. Among inpatients, a total of 24 visited the hospital for day surgery, which requires only a single day for hospitalization, surgical operation, and recovery. Of the remaining 8 inpatients, 1 was admitted to the intensive care unit. Facial features of day-surgery inpatients were registered when they arrived at the patient day surgery center (PDSC). Before entering the surgical operating room and during the operation, a member of the hospital staff confirmed the patient number for verification purposes and then used the mobile app for facial recognition. Nonday-surgery inpatients were registered the day before the operation and tracked on operation day as described for day-surgery patients. As they did not leave the hospital the same day, identification was confirmed in the recovery room after completion of surgery. A total of 32 inpatients were all pediatric patients and were aged between 1 and 17 years. Among 32 inpatients, 31 of them were unconscious in the operating room because of anesthesia. All inpatient facial data were captured when conscious before entering the operating room.

### Statistical Analysis

Total comparison trials are calculated based on one-to-one matching with the number of sequentially registered patients and each patient’s facility visits. The sum of each patient’s facility visits is considered as true value. Here, the sensitivity is the percentage of correct match of a patient at various facilities (true-positive value). The specificity is the percentage of incorrect match against all previously registered patients (true-negative value). Sensitivity and specificity are calculated as below:

Sensitivity = (True Positive) / (True Positive + False Negative) (1)

Specificity = (True Negative) / (True Negative + False Positive) (2)

## Results

### Mobile App Development

With a Samsung Galaxy Note 3, the mobile app was able to capture facial features and process facial recognition within 0.5 seconds. The minimum matching percentage of facial recognition to verify a patient was set to 95%. The minimum facial width and length between irises was set to 30 pixels. Recognizable maximum vertical and horizontal facial posture angles were 15 degrees and 40 degrees, respectively.

When verifying a patient, the newly extracted facial image is compared with the initially stored facial template. If the similarity between the 2 templates is over 95%, the facial recognition engine recognizes the images as belonging to the same patient. When recognizing a patient, the app calculates the Euclidean distances between 3-dimensionally repositioned landmarks of templates, weighing the distances of the eyes and nose. The accuracy of the facial recognition mobile app tested at Seoul National University Children’s Hospital was 99%.

**Figure 1:**
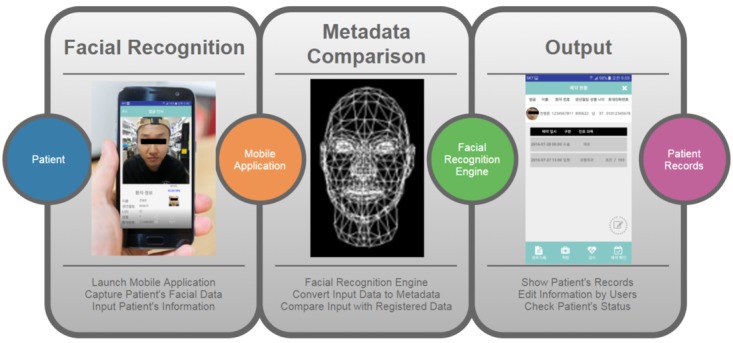
Process of facial recognition application.

The facial recognition engine supports the Android, IOS, Embedded Linux, Windows, IBM AIX, and Sun Solaris operating systems. As the facial template size is minuscule, it can be stored in a secured server, and a copy of the template can be stored in a mobile device for offline use. The overview of the facial recognition app process is as shown in Figure 1. When the patient is first enrolled, the mobile camera captures the patient’s face image, and the facial recognition engine creates and stores the facial template. Then, when patient identification is needed at another facility, the facial recognition engine captures the patient’s face image, generates a new facial template, and compares the input (newly generated facial template) with the registered data (initially stored facial template). After facial recognition successfully identifies the patient, the output shows the patient’s records, and detailed information of the patient can be edited. Detailed hospital-wide studies and design of the app are described in the section below.

### Outpatient Studies

**Figure 2:**
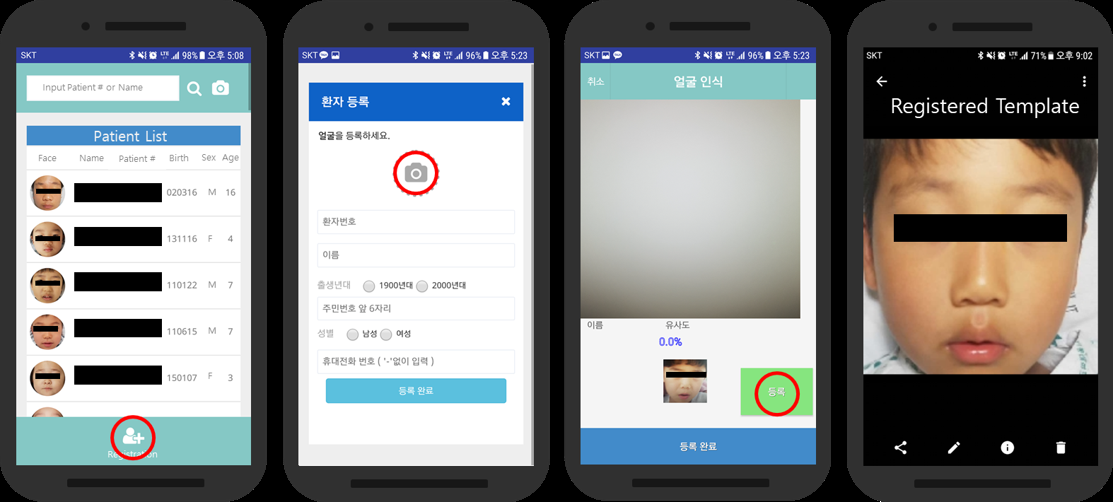
Registration of facial template.

**Figure 3:**
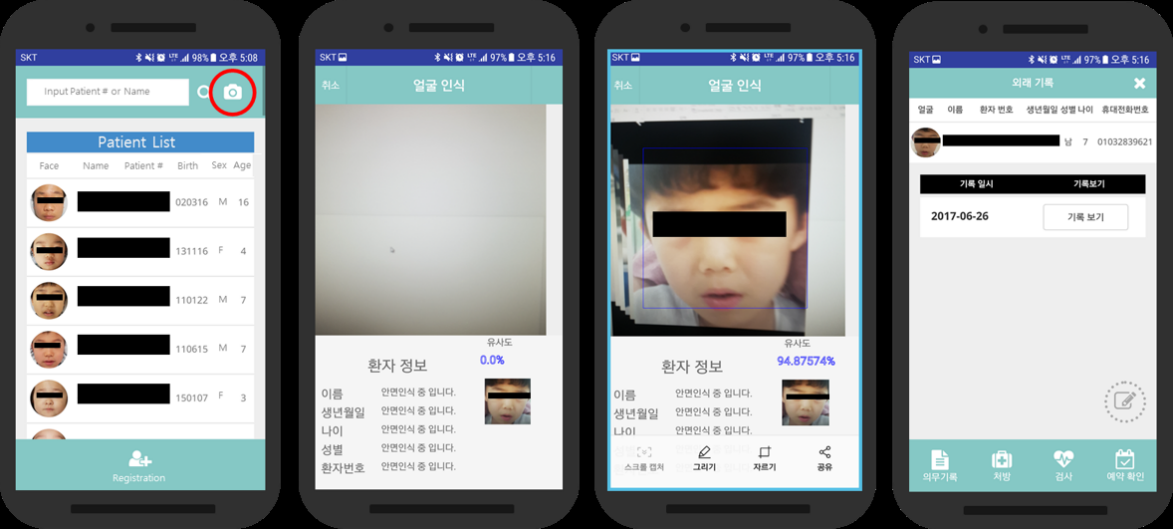
Facial recognition process.

A total of 30 hospital outpatients were registered and tracked (Figures 2 and 3). Figures 2 and 3 demonstrate the patient registration process and the facial recognition process. Registration of a patient requires an initial process of capturing the patient’s facial information. There is a registration button at the bottom of the main screen of the app (Figure 2). To register a patient’s facial data, patient number, name, date of birth, and phone number are required to be typed as shown in the second image of Figure 2. Capturing the patient’s facial data and an example of the registered patient’s facial image are shown in Figure 2.

Tracking a patient requires capturing patient’s facial image to compare with registered data. There is a camera button at the upper right corner of the main screen of the app (Figure 3). The steps involved in capturing the patient’s facial data and comparing the input with the registered data are shown in Figure 3 (see center). The last image of Figure 3 is an example screen of a successfully identified patient.

Average patient age was 5 years because of the fact that the research was done at the children’s hospital where the main plastic surgery is to correct burns, birth defects, etc. Each patient visited an average of 4 different hospital facilities and verified their identification with the facial recognition mobile app (Table 1).

**Table 1:** Number of outpatient verification trial results.

Characteristic		Pass verification, n	Fail verification, n
**Age (years)**			
	0-10	28	0
	11-20	2	0
**Sex**			
	Male	19	0
	Female	11	0
**Facility visit^a^**			
	Male	86	0
	Female	50	0

^a^Total number of different facilities where each patient was verified using both traditional methods and the facial recognition mobile app.

**Figure 4:**
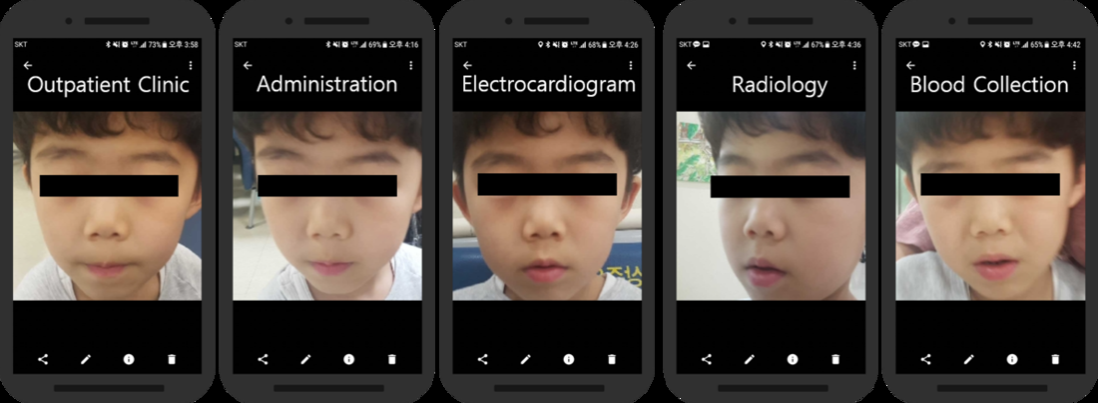
Verified outpatient's input images in various facility.

When visiting different facilities, all 30 patients were successfully verified by the facial recognition system, and the images recognized as *correct* were captured and stored separately in a secure hard drive. An example of different facility visits by an outpatient and the images captured when verifying a patient using the facial recognition system is shown in Figure 4.

**Figure 5:**
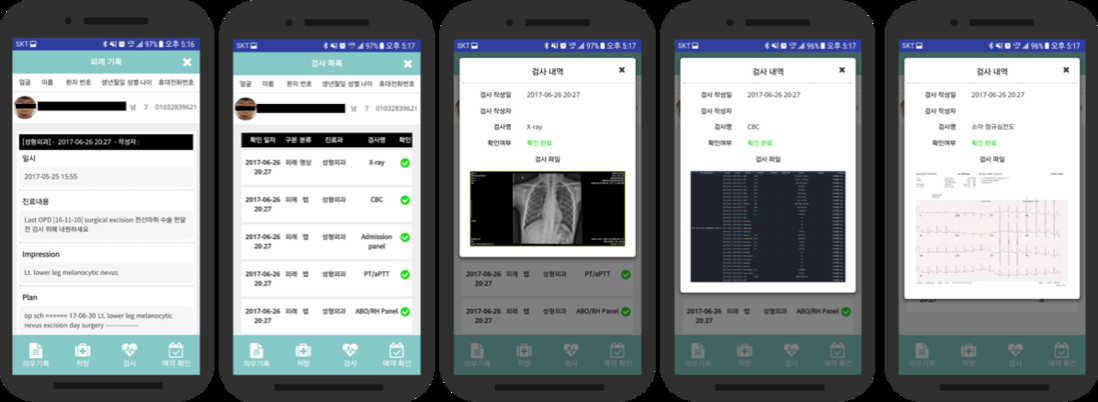
Medical recordings and examination.

Ordered patient examinations and treatments were also recorded using the app and uploaded to the database so that hospital staff could determine whether the patient had been treated or examined appropriately. As shown in Figure 5, green color indicates that the examination was successfully confirmed by hospital staff; X-ray image, complete blood count data, and electrocardiography data were shown here as an example of records.

**Figure 6:**
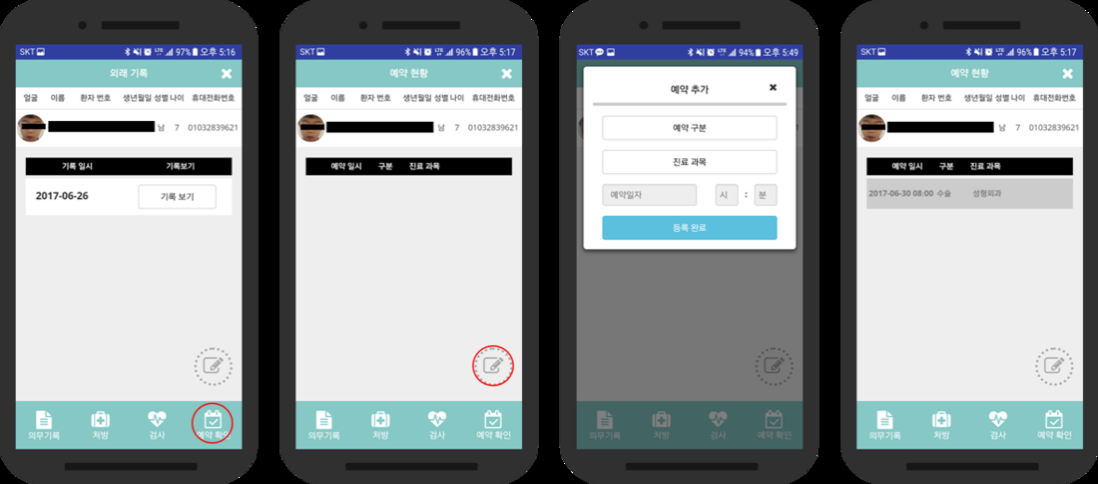
Appointment record.

Appointment records were also updated in the app to facilitate scheduling and verification of future visits. The record includes options for inpatient, outpatient, department, and the date (Figure 6). All the information recorded in the app program is compared with the EMR, and it showed that the previously recorded information can be maintained elsewhere after the facial recognition process and can be utilized in the EMR interworking system in the future.

### Inpatient Studies

A total of 32 inpatients were registered and tracked (Figures 2 and 3). Average patient age was 5 years because of the same reason stated in the outpatient section above. In the operating room, 31 out of 32 inpatients were identified with the mobile facial recognition app under anesthesia. In addition, the app was used to confirm the surgical details. During recovery, day-surgery inpatients were verified with the facial recognition mobile app and treated or prescribed in the recovery room as indicated by the app. When patients were ready to leave the hospital, their identification was confirmed via the facial recognition mobile app once again at the PDSC. Each patient was identified an average of 4 times after their facial features had been registered before entering the surgical operating room, which was recorded as a facility visit. The single unrecognizable patient was not recognized after surgery because of the compression bandage covering the patient’s face (Table 2).

**Figure 7:**
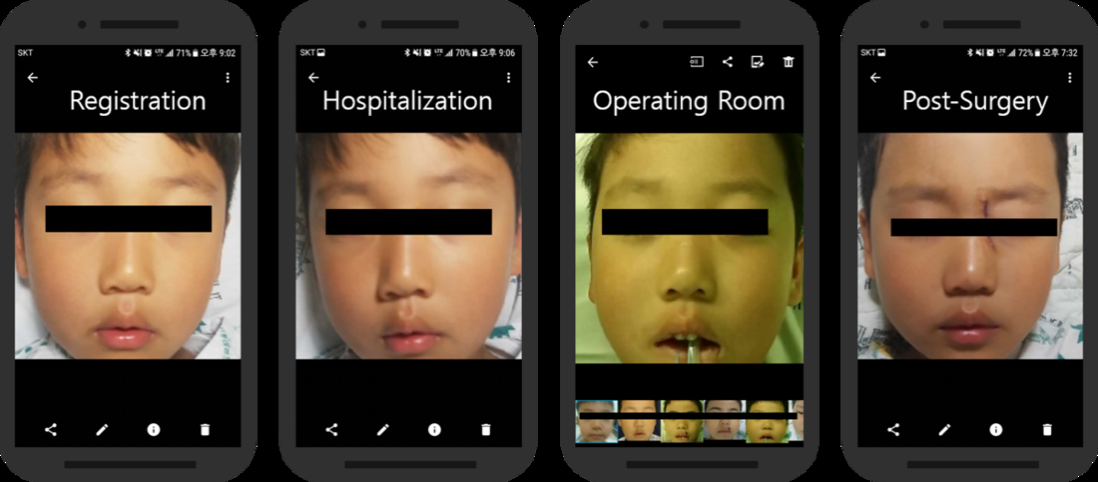
Verified inpatient's input images in various facility.

A total of 31 of 32 inpatients were recognized and verified by the facial recognition mobile app even after facial surgery. An example of different facility visits by an inpatient and the images captured when verifying a patient using the facial recognition system are shown in Figure 7.

**Table 2:** Number of inpatient verification trial results.

Characteristic		Pass verification, n	Fail verification^a^, n
**Age (years)**			
	0-10	27	0
	11-20	4	1
**Sex**			
	Male	18	0
	Female	13	1
**Facility visit^b^**			
	Male	89	0
	Female	60	1

^a^One failed verification was because of a compression bandage covering the patient’s face after surgery.

^b^Total number of different facilities where each patient was verified using both traditional methods and the facial recognition mobile app.

### Statistical Analysis

As the total number of patients is 62 and each patient’s facility visits varies from 2 to 5, the total individual comparison trials based on one-to-many matching resulted in 8899. As shown in Tables 1 and 2, the sum of each patient’s facility visits is 286. The sensitivity and specificity resulted in 99.7% (285/286) and 100.00% (8613/8613), respectively, with an accuracy of 99.99% (8898/8899).

## Discussion

### Principal Findings

Medical malpractice caused by failure in patient verification is a critical problem when considering patients’ safety, as it can lead to operations on incorrect body sites and other further treatments. It was estimated that as many as 98,000 people die every year from medical errors [20]. Thus, accurate and precise patient identification is essential before every procedure in a hospital setting [21,22]. Traditional methods for patient identification include patient verification of name, date of birth, or hospital-provided patient number. This approach is not secure enough for patient verification when considering patient safety [23,24].

To mitigate current risks to patient safety, physical objects and biometric technologies such as RFID bracelets, fingerprint scanners, and iris scanners have been employed, but each approach has important limitations. Physical objects must always be present and kept secure to successfully confirm a patient’s identity [10,25]. Fingerprint scanners may facilitate infection transmission, and iris scanners cannot be used on unconscious patients [26,27]. If these limitations are addressed, biometrics are one of the most reliable alternative options for patient verification under the minimum hospital requirements for patient verification. Facial recognition is another promising biometric approach to patient verification. It does not require any physical contact and can be used even when a patient is unconscious.

Our implementation of a facial recognition system through a mobile app resulted in 99.99% accuracy in patient verification. The accuracy of the facial recognition app would have been 100% had it not failed on a single patient whose face was covered with a compression bandage post surgery. With the exception of this case, the facial recognition mobile app was able to recognize and verify all other facial surgery patients. Moreover, patients under anesthesia in the operating room were recognizable and verified via the facial recognition mobile app.

For both patients and hospital staff, the traditional verification process of calling a patient’s name, matching the recorded patient number by eye, or confirming the date of birth is time consuming and a suboptimal use of human resources [23]. Furthermore, asking patients for their patient number, requesting them to put their finger on a fingerprint scanner, or requesting to scan their eyes with an iris scanner are impossible under circumstances that often occur in a hospital setting. In contrast, mobile facial recognition systems can verify random patients at any time simply by taking pictures of the patient (whether conscious or unconscious) in lieu of making specific requests of the patient. When patient verification is imperative during serious circumstances, such as in the operating room, facial recognition systems provide a quick and simple way to identify patients, especially because the mobile app system does not require a space-demanding and expensive device such as a scanner. Through the mobile app, facial recognition technology is a convenient and secure alternative compared with other biometrics for patient verification because the app requires only a mobile phone. Thus, the facial recognition mobile app described herein is easily accessible and an accurate patient identification tool in the hospital setting.

### Limitations

In the case of distance, there is a correlation with the size of the face captured by the camera. For the facial recognition app of this research, the camera resolution was fixed at 480×720 pixels, and it was developed considering the distance of less than 60 cm in terms of the usage form of a mobile phone. However, the distance is adjustable, and if the size of the face detected on the screen exceeds 120×120 pixels, face recognition is possible. For example, if we apply the developed app to the interactive advertisement of New York Times Square, the app can recognize the faces of people 70 m away by using a high-resolution zoom camera and filtering out participants. As the example mentioned, distance is adjustable with a better camera, but at the same time, it requires greater amount of the central processing unit (CPU) resources for facial recognition when the image resolution is high. Thus, as the CPU of the mobile phone is slower than the personal computer, we aimed at recognizing the face within 0.5 seconds by fixing it at 480×720 resolution in this research.

As other studies on facial recognition stated, varying levels of light decreases the accuracy of the facial recognition process [18]. Currently, our facial recognition system is also limited by lighting. When the registered facial features were recorded under a bright light, patient recognition in darker places or a place where the light entered from a different angle, creating shade, resulted in delayed verification requiring up to 5 min for light correction to match images in the database. In addition, it was not possible to link the system to the EMR because the app was developed for test purposes only.

### Comparison With Prior Work

Previous researches have implemented facial recognition technology for various purposes with a main focus on identifying an individual. However, one of the limitations is that it requires specific sensors and motions to be used in patient verification [18]. In a hospital setting, the identification process should be simple and effective, as patients could be in various conditions such as in an unconscious state. The facial recognition mobile system is effective and simple as it only requires a mobile phone and does not require specific motion such as turning and tilting the head in various directions for patient identification.

The other limitation of the facial recognition research conducted is the cost of processing facial recognition. Other research revealed that facial images derived from CT scans can be accurate [19]. However, the process of extracting the facial features of a patient and the cost of CT scans are not adequate for patient verification purpose. Even though CT scans are higher resolution compared with a mobile camera, simplicity and cost-effectiveness of the mobile facial recognition system are more suitable for the patient verification process.

### Future Research

Further research is necessary to resolve the light sensitivity suffered by the app and to more accurately evaluate its performance for patient verification in the hospital, as well as to link the app with the hospital EMR to improve accessibility by hospital staff. Moreover, if a hospital-wide stationary camera-based facial recognition system is developed as a patient verification method, it could be used as a surveillance camera to monitor and verify patients entering various hospital facilities.

### Conclusions

The facial recognition mobile app described here has been developed for patient verification purposes. The developed app contains 5 main parts suited for hospital usage: registration, medical records, examinations, prescriptions, and appointments. Hospital staff registered the facial feature of both outpatients and inpatients in the facial recognition database. The implementation of the facial recognition mobile app in the hospital setting proved a suitable alternative patient verification method with an accuracy of 99%. Once the facial recognition system is fully linked to EMR, it will be fully accessible to clinics and hospitals for patient verification.

## Abbreviations

CPU: central processing unit
CT: computed tomography
EMR: electronic medical record
IRB: Institutional Review Board
NDK: Native Development Kit
PDSC: Patient Day Surgery Center
RFID: radio-frequency identification
